# Brain connectivity associated with cascading levels of language

**DOI:** 10.15761/JSIN.1000139

**Published:** 2016-11-10

**Authors:** Todd Richards, William Nagy, Robert Abbott, Virginia Berninger

**Affiliations:** 1 Integrated Brain Imaging Center, Department of Radiology, University of Washington, Seattle, USA; 2 School of Education, Seattle Pacific University, Seattle, USA; 3 Statistics and Measurement, University of Washington, Seattle, USA; 4 Learning Sciences and Human Development, University of Washington, Seattle, USA

**Keywords:** language impairment, dyslexia

## Abstract

Typical oral and written language learners (controls) (5 girls, 4 boys) completed fMRI reading judgment tasks (sub-word grapheme-phoneme, word spelling, sentences with and without spelling foils, affixed words, sentences with and without affix foils, and multi-sentence). Analyses identified connectivity within and across adjacent levels (units) of language in reading: from subword to word to syntax in Set I and from word to syntax to multi-sentence in Set II). Typicals were compared to (a) students with dyslexia (6 girls, 10 boys) on the subword and word tasks in Set I related to levels of language impaired in dyslexia, and (b) students with oral and written language learning disability (OWL LD) (3 girls, 2 boys) on the morphology and syntax tasks in Set II, related to levels of language impaired in OWL LD. Results for typical language learners showed that adjacent levels of language in the reading brain share common and unique connectivity. The dyslexia group showed over-connectivity to a greater degree on the imaging tasks related to their levels of language impairments than the OWL LD group who showed under-connectivity to a greater degree than did the dyslexia group on the imaging tasks related to their levels of language impairment. Results for these students in grades 4 to 9 (ages 9 to 14) are discussed in reference to the contribution of patterns of connectivity across levels of language to understanding the nature of persisting dyslexia and dysgraphia despite early intervention.

## Introduction

The current interdisciplinary study drew on contributions from developmental psycholinguistics and neuroimaging to study the multiple levels (units) of language in the reading brain. Developmental psycholinguistic research has shown that there is a progression from sub-word sounds to spoken single words to two-word combinations to multi-word syntax in clauses to multi-syntax/clausal constructions in oral language development [1]. Each successive developmental milestone draws on a higher level (unit) of oral language. Relatively less research has focused on how the brain inter-relates the various levels-of-language involved in learning to read so that the sub-word level is coordinated with the word-level, the word-level is coordinated with the syntactic-level, and the syntactic-level is coordinated with the text-level in the reading brain. Thus, the goal of the current study was to investigate how the brain supports each of the increasingly larger units (levels) of language for orchestration of mind [2] in constructing a reading brain. Of interest was both those who learned to read without a struggle and those who experienced persisting struggles during middle childhood and early adolescence in acquiring language skill at specific levels-of –language such as word spelling in dyslexia [3] or syntactic/text levels for listening and reading comprehension and oral and written expression in specific language impairment (SLI) [4,5] also referred to as Oral and Written Language Learning Disability (OWL LD) [6].

FMRI functional connectivity from specific regions of interest (ROI) for tasks at each level of language was used to study how the brain may draw on common as well as unique brain connectivity across adjacent levels of language. The amount of connectivity detected with other brain regions from a given seed, even after controlling for multiple comparisons [7], may be surprising to some. The brain atlas often used in connectome research [8-11] and also used in this fMRI connectivity study—the Jülich histological (cyto-and myeloarchitectonic) atlas [12,13] provides much more fine-grained detail and increases the number of potential connections compared to prior atlases with fewer locations within regions.

**Figure 1:**
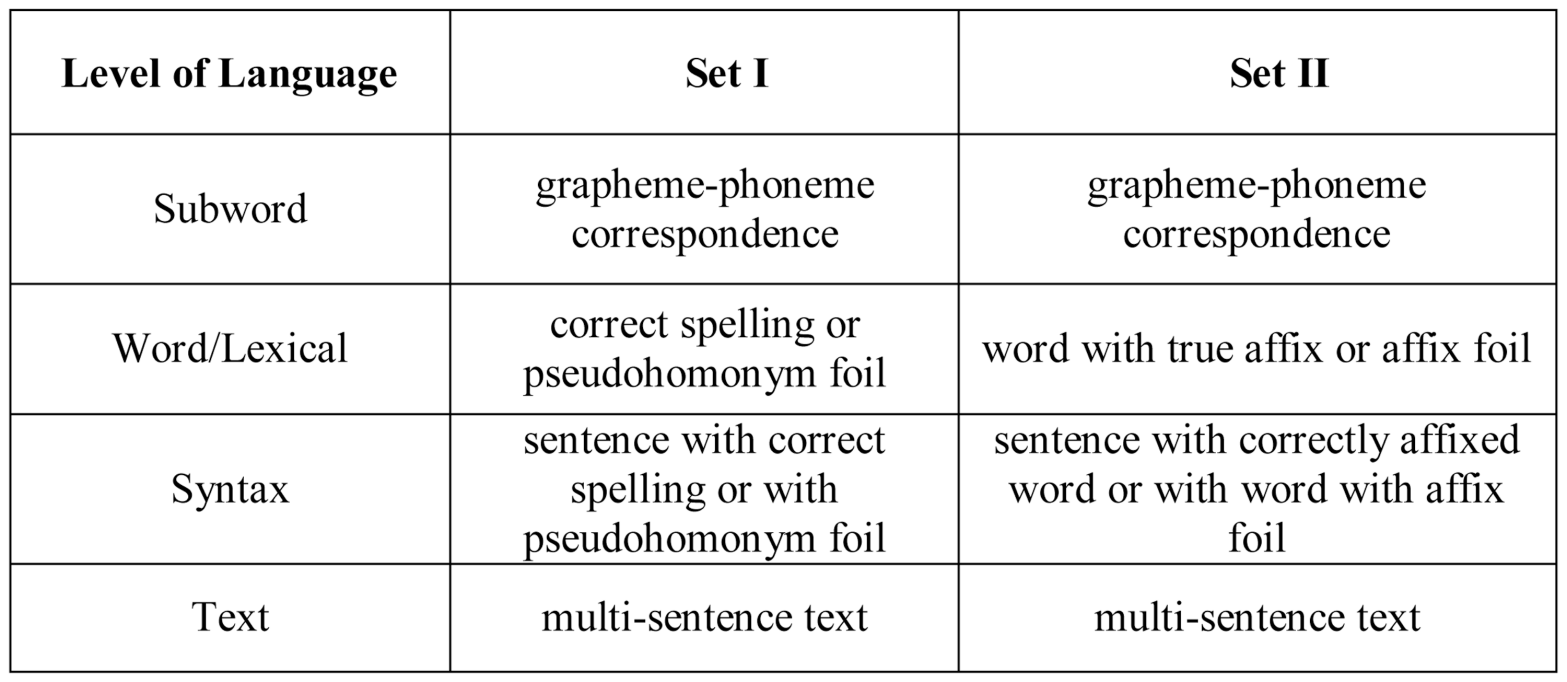
Set I and Set II Comparisons across Four Levels of Language in Reading Brain. Bolded word and syntax levels contrast across Sets I and II in linguistic properties of words with and without foils. Unbolded subword and text are constant across Sets I and II.

As shown in Figure 1, two sets of fMRI tasks were used to study common and unique connectivity across adjacent levels of language. The Set I fMRI tasks required deciding if word pairs were correctly spelled words or were pseudohomophone foils—pronounced the same as a real word but not spelled correctly (word-level task); and if sentences with and without such homonym foils were meaningful (a syntax-level task). Set II fMRI tasks required deciding if word pairs did and did not have true affixes (a word-level task) and if sentences with and without words with affix foils were meaningful (a syntax-level task). For both Sets, word level tasks were compared to a common sub-word level task (deciding if a one- or two- letter grapheme corresponds to the same phoneme); and the sentence level tasks were compared to a common text-level task (reading multiple sentences and deciding if the conclusion at the end, which requires inferential thinking, was true or false). Reading comprehension also draws on communication across the language and cognitive systems—both at the word- level for vocabulary [14] and sentence- and text- levels for passage comprehension [15].

Also, the two sets of tasks used for the word-level and the syntax-level were designed to evaluate how linguistic features of words and their syntactic context might affect connectivity when level-of-language was kept constant. For Task 2 in Figure 1, deciding whether a word is a correctly spelled real word versus a pseudohomonym foil (pronounced the same but not spelled correctly for a real word with a specific meaning) requires knowledge beyond permissible grapheme-phoneme correspondences; to make this decision, the reader must integrate orthographic and phonological patterns and semantic knowledge for the whole lexical unit [16-18]. Consider sammon and salmon, both of which are pronounced the same, but only one is a correctly spelled word with meaning.

In contrast, for Task 4 in Figure 1, because English is a morphophonemic orthography [19,20] the task is to decide whether common spelling units are functioning as true affixes or not [21,22]. Consider summer and swimmer; both have a common spelling pattern which is a true suffix only in the second word. A true morpheme at the end of a word transforms a word base as to tense, number, or grammar [23] and may in some instances change the phonology of the base word [24]; or if at the beginning of a word, a true morpheme (prefix) qualifies meaning of a base word [25]. Consider the –al in national, which transforms a noun nation into an adjective, but also changes the pronunciation of the base word nation; and consider the word international in which the prefix transforms the meaning of nation to mean beyond a single nation. The presence of these “fixes” on words in a sentence can also affect whether a sentence is meaningful, depending on whether the affixed word fits the context of the sentence syntax.

Thus, the goal was pattern analyses for connections shown to be of statistically significant magnitude rather than testing the significance of the difference in mean magnitude for a particular connection. The first hypothesis tested was derived from the evidence-based cascading levels-of -language theoretical framework, according to which the next higher level-of-language shares connections in common with the immediately adjacent lower level-of -language but also exhibits connections unique to that next higher level-of-language [26].

To test the first hypothesis, connectivity was measured from each of four seeds. The first seed—left precuneus—has been shown in fMRI ROI brain imaging studies to be involved in the orthographic coding of word-specific spellings underlying both reading and writing during middle childhood and early adolescence [7,27]. Precuneus which has been shown to be part of a central neocortical hub known as the rich club because it participates in many functional systems and plays a key role in integration across the neural networks [11], may be involved within and across levels of language in the multi-leveled reading system. The other three seeds were identified in a meta-analysis of brain processing of written words [28] and brain research on reading: left occipital temporal [29], left supramarginal [30,31], and left inferior frontal [31,32]. The second tested hypothesis was that students with persisting problems in learning to read and spell words (dyslexia) or process the morphology of the written words in the language (OWL LD) show different patterns of connectivity from these seeds across adjacent levels of language in the multi-leveled reading system in comparison to each other and to those who are typical language learners and learn to read without a struggle.

## Methods

### Participants

Participants were recruited from another study in the same research center that had administered a comprehensive test battery to students in grades 4 to 9 using procedures described in Berninger, et al. [26]. Those who met research criteria for typical oral and written language learners (controls) or dyslexia or OWL LD, were right handed, and did not wear metal braces or other non-removable metal were invited to participate in the brain imaging study.

### Sample characteristics

All participants were in the age range of 10 to 14 and were of European-American ethnicity; and their biological or adoptive parents had at least some postsecondary education or a college degree. Assignment to diagnostic group (typical, dyslexia, or OWL LD) was based on current scores on normed measures in the test battery for handwriting, word reading and spelling, and oral receptive and expressive syntax, and developmental and educational history (current and past) obtained from questionnaires parents completed while their children were formally tested.

Inclusion criteria for typical group were (a) no indicators of a specific learning disability (SLD) in handwriting, word reading or spelling, or listening or reading comprehension or oral or written language expression, (b) a Verbal Comprehension Index score within the normal range (80 or higher but most were 90 and above and even above the population mean of 100), and (c) parental reported developmental history prior to kindergarten and educational history beginning in kindergarten and continuing into the present of no oral and/or written language learning difficulties. Altogether 4 males and 5 females in grade 5 (n=1), grade 6 (n=2), grade 8 (n=3), or grade 9 (n=3) (age range 10 to 14) qualified for participation in the brain imaging study as typical.

Altogether 10 males and 6 females in grades 4 (n=3), 5 (n=1), 6 (n=5), 7 (n=4), 8 (n=1),and 9 (n=2) qualified for the dyslexia group based on at least two word reading or spelling skills being below -2/3 SD (25^th^ %tile) and no indication of listening comprehension or oral comprehension difficulties currently or in the past, but a past and current history of persisting problems with word reading/decoding and word spelling/encoding [3]. Altogether 2 males and 3 females in grades 4 (n=1), 5 (n=1), 6 (n=1), and 7 (n=2) qualified for the OWL LD group based on at least two listening comprehension, reading comprehension, oral expression, and/or written expression skills being below -2/3 SD (25^th^ percentile), a preschool history of struggling with oral language learning, and current and past history of struggle with listening comprehension, reading comprehension, oral expression, and/or written expression since kindergarten.

### Assessing achievement

The normed measures in the test battery were based on raw scores transformed into scaled scores (M=10, SD=3) or standard scores (M=100, SD=15) based on national standardization norming samples or transformed into z-scores for grade (M=0, SD=1) based on large research samples. See Table 1 for summary of group Means and SD's for each measure.

### Handwriting measures

On the Alphabet Writing Task (Alphabet 15z), an experimenter-designed test, children are asked to handwrite in manuscript (unjoined letters) the lower case letters of the alphabet from memory as quickly as possible in alphabetic order, but to make sure others can identify the letters. The raw score is the number of letters that are legible and in correct order during the first 15 seconds. The raw score is converted to a z-score (M=0, SD=1), based on research norms for grade (inter-rater reliability .97). On the Detailed Assessment of Speed of Handwriting (DASH) the DASH Copy Best and the DASH Copy Fast [33] the task is to copy a sentence with all the letters of the alphabet under contrasting instructions: one's best handwriting or one's fast writing (interrater reliability .99). Students can choose to use their usual writing— manuscript (unconnected) or cursive (connected) or a combination. The scaled score (M=10, SD=3) is based on legibility for single letters in copied words within 2-minute time limit. In the current study, two testers reviewed all the scored handwritten measures to reach consensus on scoring.

#### Word reading and spelling measures

For the Test of Silent Word Reading Fluency (TSWRF) (test-retest reliability is .92) [34], the task is to mark the word boundaries in a series of letters arranged in rows. The standard score (M=100, SD=15) is the number of correctly detected and marked word boundaries in 3 minutes, which is a measure of timed single word reading. A subtest of the Test of Orthographic Competence (TOC) [35], which yields a scaled score (M=10, SD=3), was given. For the TOC Word Choice (Homophone Choice ages 9 to 12 or Word Choice ages 13 to 16) (test-retest reliability .72 to .75), the task is to identify a correct spelling for a specific word and not confuse the correct spelling with a homophone foil that is pronounced the same but not spelled the same as a real word; thus this measure is a behavioral analogue of the fMRI word-specific spelling Task 2 used in the current study. For the TOC Letter-Choice subtest (test-retest reliability .84 to .88), the task is to choose a letter in a set of four provided letters to fill in the blank in a letter series to create a correctly spelled real word (word-specific spelling); thus, this measure is a behavioral analogue of the fMRI writing task for written words underlying reading and spelling used in Richards, et al. [7].

For the experimenter-designed Comes From z, which is a measure of morphological word form storage and processing, the task is to judge whether or not a read word is derived from a base word [21]. Example items follow: Does corner come from corn? Does builder come from build? In both cases the words in a pair contains a common spelling (er), but it may or may not function as a morpheme that transforms a base word. Raw scores are transformed to z-scores (M=0, SD=1) based on research norms for elementary and middle school grades. Thus, this measure is a behavioral analogue of the fMRI affixed word reading task 4.

### Oral language measures

WJ III Oral Comprehension [36], which is an aural cloze task requires supplying a word orally during pause in unfolding oral text and yields a standard score (M=100, SD=15) (test-retest reliability .88). Also given was Clinical Evaluation of Language Function 4^th^ Edition CELF IV [37] Formulated Sentences (CELF IV Form Sent), which requires constructing an oral sentence from provided words and yields scaled scores (M=10 and SD= 3) (test-retest reliability .62 to .71).

### Written language measures

WJ III Passage Comprehension [36] (test-retest reliability is .85), a reading comprehension analogue of the oral cloze task, was also given which requires supplying orally a missing word in the blank that fits the accumulating context of the sentence and preceding text. The WJ III Writing Fluency [36] (test-retest reliability .88) was also given on which the task is to compose a written sentence for each set of three provided words, without changing them in any way. There is a 7 minute time limit, for this measure which is sensitive to syntax construction ability. For both measures the score is a standard score (M=100, SD=15).

### Cognitive oral language translation measure

The Wechsler Intelligence Scale for Children, 4^th^ Edition (WISC IV) [38] Similarities, Vocabulary, and Comprehension subtests were given to obtain a Verbal Comprehension Index Score (WISC IV VCI) which is a standard score with M=100 and SD=15 (test-retest reliability .93 to .95). The tasks require using oral language to express cognitions.

### fMRI Reading tasks during scanning

An fMRI connectivity design was used instead of a block design that makes comparisons across repeating conditions. Connectivity scores were derived from each of the four seed points on each of the six multi-leveled reading tasks. The reading tasks were all programmed, timed, and coordinated with the scanner triggers using E-prime and in-house LabView software. All tasks were taught and practiced outside the scanner before performing them during scanning. A score of 90% correct on training tasks was required prior to participating in scanning. Participants practiced lying still before entering the scanner and were instructed to lie still throughout the scanning. They also practiced the reading tasks before scanning and had to achieve 90% accuracy on them to continue participation to ensure that the brain imaging did not reflect inability to do a task. During the functional scans, they were also instructed to look at a fixation cross (no reading task; 180 time points) or to complete a specific reading task. To ensure continuous cognitive engagement, each reading task was presented with self-paced advancing of stimuli for two minutes; 960 timepoints).

#### 

##### Reading task 1

Subword grapheme-phoneme judgments. Each pair is constructed from a single letter or letter group. The participant is instructed to think about the small sounds that could go with each pair of single letters, a single letter and a letter group, or letter groups and then press yes if each letter and/or letter group in a pair presented on the screen can stand for the same sound or no if cannot stand for the same sound. Example of yes pair is “c s”. Example of no pair is “d” and “m”.

##### Reading task 2

Lexical judgments linked to identifying correct word-specific spellings among homonym foils. The participant is instructed to press yes if written word on screen is a correctly spelled real word, but press no if written word on screen is not a correctly spelled word, even though when pronounced it sounds like a real word. Example of yes item is “bus.” Example of no item is “eer.”

##### Reading task 3

Syntactic judgments with and without homonym foils. The participant is instructed to press yes if the sentence could be a real sentence that is meaningful because all the words are spelled correctly and make sense in the sentence, but press no if the sentence is not meaningful because all the words do not make sense in the sentence. Each sentence was presented for 3 seconds. The “no” items differed from the “yes” items by only one word which was a homonym foil. This is an example of a yes sentence: “The bee, which buzzes, can sting you.” This is an example of a no sentence: “The bee, witch buzzes, can sting you.”

##### Reading task 4

Lexical judgments about true affixes among non-affix foils. The participant is instructed to press yes if the word has true affix, but to press no if the word has the same spelling as an affix but is not an affix. This is an example of a yes item: untie. This is an example of a no item: under.

##### Reading task 5

Syntax judgments with and without affixed foils. The participant is instructed to press yes if the bolded word could fit the sentence and the sentence is meaningful. This is an example of a yes item: He was unfit physically. This is an example of a no item: He was unfitted physically.

##### Reading task 6

Multi-sentence text judgments. The participant is instructed to read each of the four sentences that will appear on the monitor one at a time and then press yes if the fifth sentence is true based on the four prior sentences read or no if it is false. Five written sentences are presented on the monitor one at a time (each presented for constant time interval). The last one is always a statement about the accumulating text so far that can be answered true (yes) or false (no).

**Example set for a true response follows:**

**Sentence 1**: John handed Bill a note.
**Sentence 2**: It was from Sarah.
**Sentence 3**: Sarah had written that she wanted to talk to Bill.
**Sentence 4**: Bill frowned when he read the note.
**Sentence 5**: True or False? (press key to answer) (True)
Bill was not pleased with what Sarah had written.

**Example set for a false response follows:**

**Sentence 1**: Tomorrow is the day of the picnic.
**Sentence 2**: If it rains, the picnic will be cancelled.
**Sentence 3**: Amy listens to the weather report.
**Sentence 4**: She hopes it will rain.
**Sentence 5**: True or False? (press key to answer) (false)
Amy wants to go to the picnic.

## MRI Data acquisition

Functional magnetic resonance imaging (fMRI) connectivity scans were obtained on a Philips 3 T Achieva scanner (release 3.2.2 with the 32-channel head coil). All scans were acquired at the Diagnostic Imaging Sciences Center in collaboration with the Integrated Brain Imaging Center and had Institutional Review Board approval. Each participant was screened for MRI safety before entering the scanner. Physiological monitoring was performed using the Philips pulse oximeter placed on the left hand index finger for cardiac recording; and respiration was recorded using the Philips bellows system where the air-filled bellows pad was placed on the abdomen. Head-immobilization was aided by using an inflatable head-stabilization system (Crania, Elekta).

Scanning included the following MRI series: 1) 3-plane scout view with gradient echo pulse sequence: TR/TE 9.8/4.6 ms; Field of view 250 × 250 × 50 mm; acquisition time 30.3 s; 2) reference scan (used in parallel imaging) with gradient echo pulse sequence: TR/TE 4.0/0.75 ms; Field of View 530 × 530 × 300 mm; acquisition time 44.4 s; 3) fMRI scan with echo-planar gradient echo pulse sequence (single shot): TR/TE 2000/25 ms; Field of view 240 × 240 × 99 mm; slice orientation transverse, acquisition voxel size 3.0 × 3.08 × 3.0 mm; acquisition matrix 80 × 80 × 33; slice thickness 3.0, SENSE factor in the AP direction 2.3; epi factor 37; bandwidth in the EPI frequency direction 1933 Hz, SoftTone factor 3.5, sound pressure 6.1 dB, 180 dynamic scans; 5 dummy scans; fold over direction AP, 396 dynamic scans; 4) B0 field map imaging with gradient echo pulse sequence and 2 echos; TR/TE 11/6.3 ms; delta TE 1.0 ms; slice orientation transverse, Field of view 240 × 240 × 129 mm; voxel size 1.5 × 1.5 × 3.0 mm; acquisition matrix 160 × 160 × 43, output image magnitude and phase, acquisition time 2:29 min/s; 5) MPRAGE structural scan: TR/TE 7.7/3.5 ms, Field of view 256 × 256 × 176 mm, slice orientation sagittal, voxel size 1 × 1 × 1 mm, inversion pulse delay 1100 ms, Sense factor 2 in the AP direction, acquisition time 5:33 min/s.

## fMRI Connectivity map

An fMRI connectivity map for reading was generated for each individual using four seed points in the left precuneus cortex PCC (MNI -6,-58,28 mm, Jülich atlas label GM_Superior_parietal_ lobule_7a_L), in the left occipital temporal cortex OTC (MNI -50,-60,-16 mm, between Jülich atlas labels GM_Visual_cortex_V4_L and WM_OptiC_radiation_L). in the left supramarginal gyrus SMG (MNI -52,-32,34 mm, Jülich atlas label GM_Inferior_parietal_lobule_PF_L). and in the left inferior frontal gyrus, IFG (MNI -52,20 34 mm, Jülich atlas label GM_Broca's_area_BA44_L).

Functional images were corrected for motion using FSL MCFLIRT [39], and then high-pass filtered at sigma = 20.83. Motion was also monitored in real time during scanning by observing the real-time reconstruction display of each fMRI volume on the scanner console. Motion scores (as given in the MCFLIRT report) were computed for each participant and average motion score (mean absolute displacement) for each of the groups: control 1.31 ± 1.37 mm, dyslexic 1.47 ± 1.03 mm, and OWL LD 1.32+/- 0.638 mm. Spikes were identified and removed using the default parameters in AFNI3s 3dDespike. Slice-timing correction was applied with FSL3s slicetimer and spatial smoothing was performed using a 3D Gaussian kernel with FWHM = 4.0 mm. Time series motion parameters and the mean signal for eroded (1 mm in 3D) masks of the lateral ventricles and white matter (derived from running FreeSurfer3s reconall on the T1-weighted image) were analyzed. Co-registration of functional images to the T1 image was performed using boundary based registration based on a white matter segmentation of the T1 image through epi_reg in FSL. The MPRAGE structural scan was segmented using FreeSurfer software; white matter regressors were used to remove unwanted physiological components.

## Data analyses

For Group analyses, Oxford's fMRIB software library (FSL) randomize, which performs permutations and threshold-free cluster enhancement, was used to control for multiple comparisons. The threshold-free cluster enhancement method controls for the family-wise error rate so that if p-values less than 0.05 are accepted, the chance of one more false positive occurring over all space is no more than 5%. The group statistical images were further controlled for false positives by setting a high threshold of 6.0 for the tscore tstat images produced by FSL's software randomize. A global design matrix was used as part of the GLM model in software randomizes to make the group statistical maps as described by FSL. Group maps for fMRI functional connectivity were generated for the four different seed points in the left precuneus cortex PCC, in the left occipital temporal cortex OTC, in the left supramarginal gyrus SMG, and in the left inferior frontal gyrus, IFG Broca's area for each of the 6 reading tasks. fMRI time-series were averaged within regions of interest (ROIs) formed from a 15 mm sphere centered at each seed. The averaged time-series at each ROI was correlated with every voxel throughout the brain to produce functional connectivity correlation maps, converted to z statistics using the Fisher transformation. These group maps show where in the brain there was significant functional connectivity from the seed point to other regions in the brain.

A regional analysis was used with custom software (written in FORTRAN) which was able to identify and quantify the brain regions which were significantly connected to the seed point. The Jülich histological (cyto-and myeloarchitectonic) atlas [12,13] is part of this software. Although the Jülich atlas contains many important language-related brain regions, it does not contain a specifically named region for the angular gyrus which is important for the functional reading brain [40]. However, the Jülich atlas does contain the inferior parietal lobule and its sub-parts which overlap with the angular gyrus.

For the typical language learners, fMRI connectivity within and across adjacent levels of language was analyzed for each of the six levels of language tasks (separately for Set I and Set II) to identify both common and unique connectivity across adjacent levels of language. However, for the dyslexia group, these analyses were conducted only for the tasks that correspond to the hallmark impairments in dyslexia—subword grapheme phoneme correspondences (Task 1) and word-specific spelling (Task 2) related to both word reading and word spelling in Set I. For the OWL LD group, these analyses were conducted only for the tasks that correspond to the hallmark impairments in OWL LD—morphology (Task 4) and syntax (Task 5) in Set II. A sentence reading comprehension task is especially sensitive to the reading comprehension problems of OWL LD (SLI) [6, 41].

## Results

**Figure 2:**
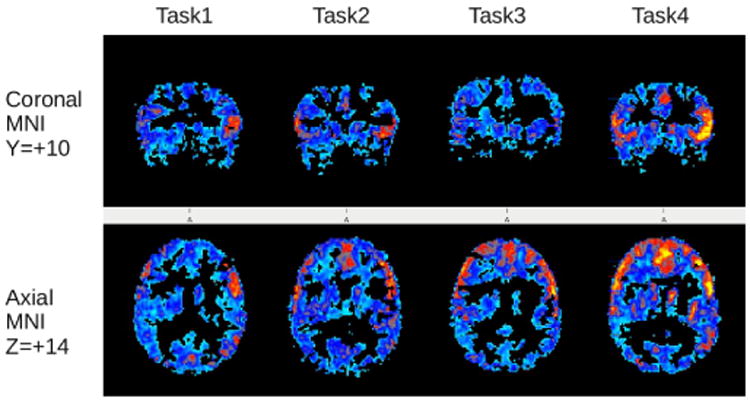
Changing profiles of connectivity across cascading levels of language. Statistical fMRI connectivity group tscore maps showing extent of significant brain regions connected to Broca's area during 4 different reading tasks corresponding to different levels language for typical readers. Task1= subword Grapheme task, Task2= Word level Homophone task, Task3= homonym sentence task, Task4= Multi-sentence judgement task. The red/yellow Colors indicate the significant tscores with yellow being the highest.

Inspection of fMRI brain connectivity across the fMRI tasks assessing each of four cascading levels-of-language showed that brain connectivity was not the same for each of the four levels of language and connectivity changed across the levels of language in the Set I and Set II tasks completed by the typical language learners. Figure 2 illustrates these results from the inferior frontal seed for the Set I tasks. In the text that follows common and unique connectivity results are reported from each of the four seeds first for the typical language learner group across each adjacent level of language and then for the dyslexia group and the OWL LD group for the adjacent levels of language on which they are impaired. These verbal summaries, based on all locations in the atlas used (R=Right L=Left in place sequentially until change noted) illustrate the sizable connectivity within levels of language and across adjacent levels of language. See Table 2 for the more traditional presentation of area, volume, and coordinates for the connectivity of greatest magnitude within a region for a particular seed.

## Common and unique connectivity within and across levels of language in control group

Grapheme-Phonemes versus Word Homonyms: From Left Precuneus, Common: inferior parietal lobule (R & L Pga and PGp), superior parietal lobule (R 5C2, R & L 5M,7a,7m, L 7p), visual cortex (R & L BA17, BA18, V5), callosal body, R & L cingulum, R cerebellum V; Unique to Word Level: superior parietal lobule (R 7p), L cerebellum V and VI, Broca's (L BA45), R & L Optic Radiation; and Unique to Subword Level: L superior parietal lobule (5ci), and L cerebellum V, vermis VI, and R Cerebellum VI).

From Left Occipital Temporal, Common: Broca's (R BA 44), superior parietal lobule (L7p), visual cortex (R & L BA 17BA 18, V3V, V5, L V4), cerebellum (R & L V, vermis VI, R VI), L corticospinal tract; Unique to Word Level: superior parietal lobule (R 7p and 7a), occipital (R V4, L VI, R & L optic radiation), bilateral premotor (BA6); and Unique to Subword Level: superior parietal lobule (R & L 5ci and 5M, L 7a, L 7M), and inferior parietal lobule (R & L PGa).

From Left Supramarginal, Common: inferior parietal lobule (R & L PF, L PFt), primary somatosensory cortex (L BA1, BA2, BA3b), secondary somatosensory cortex (R & L OPI), premotor cortex (R BA6), insula (R Ig2); Unique to Word Level: inferior parietal lobule (L PFcM, PFop, R & L PFM), premotor cortex (L BA6), and insula (R & L Id1), anterior intra-parietal sulcus (L hIP2), secondary somatosensory cortex (L OP3, R & L OP2 and OP4), superior parietal lobule (R & L 5Ci, 5M), visual cortex (R & L BA 17, BA18, R V5), Broca's (R & L BA 44 and BA45), primary auditory cortex, (R TE1.0, and TE1.1, R & L TE1.2), R & L cingulum, L corticospinal tract, R & L cerebellum V; and Unique to Subword Level: primary motor cortex (L BA4a1), superior parietal lobule (R & L 5ci), and insula (R Ig2).

From Left Inferior Frontal, Common: Broca's (L BA44, R & L BA45), visual cortex (L BA 17); Unique to Word Level: Broca's (R BA 44), visual cortex (R & L BA18, R BA17, LV5, primary auditory cortex (R TE1. 0, R & L TE1.2), secondary somatosensory cortex (R OPI, OP2, L OP4), inferior parietal lobule (L Pga), callosal body, L cingulum; and Unique to Subword Level: none.

Grapheme-phonemes versus affixed words. Left Precuneus, Common: inferior parietal lobule (R & L Pga, PGp), superior parietal lobule (R 5ci, R & L 5 M, 7a, 7M, L7p), visual cortex (R & L BA17 and BA 18), callosal body, R & L cingulum, R cerebellum V; Unique to Word Level: inferior parietal lobule (L PF, R PFM), visual cortex (R & L V3V, V5, LV4), secondary somatosensory cortex (R OP1, OP4), L optic radiation; and Unique to Subword Level: superior parietal (L 5ci) and cerebellum (L V, vermis VI, R VI).

Left Occipital Temporal, Common: visual cortex (R BA17, L V4, L V5); Unique to Word Level: superior parietal lobule (R 7p), premotor cortex (L BA6); and Unique to Subword Level: Broca's (R BA44), visual cortex (L BA17, R & L BA18), L superior parietal (7p), and cerebellum (R & L V, vermis VI, R V1).

Left Supramarginal, Common: primary somatosensory (L BA1, BA2, BA3b), secondary somatosensory (R & L OP1), primary motor (L BA4a), inferior parietal lobule (R & L PF, L PFt), superior parietal, (R & L 5 ci, 5M), premotor cortex (R BA6), insula (R Ig2); Unique to Word Level: primary somatosensory (R BA1, BA3b), secondary somatosensory (R & L OP4, R OP2), superior parietal lobule (R & L 7a and 7p), premotor cortex (L BA6), insula (L Ig2, Id1), visual cortex (R BA17, L V4, V5), R & L cingulum; and Unique to Subword Level: none.

Left Inferior Frontal, Common: Broca's Area (L BA44, R & L BA45). Unique to Word Level: Broca's Area (R BA44), R auditory (TE1.2), Primary somatosensory (R BA3b), secondary somatosensory (R OP2, OP3, R & L OP4), premotor cortex (L BA6), R inferior occipital-frontal fascicle; and Unique to Subword Level: visual cortex (L BA 17).

## Common connectivity across word and syntax levels and unique to these levels

### Word versus syntax levels with homonyms

Left Precuneus, Common: inferior parietal lobule (R Pga, PGp), superior parietal lobule (R 5Ci, R & L 5M, 7a, 7m, L7p), visual cortex (R & L BA17, BA18), callosal body, R & L cingulum; Unique to Syntax Level: superior parietal lobule (L 5ci), Broca's (L B45), premotor (R BA6); and Unique to Word Level: inferior parietal lobule (L gpa and PGp), bilateral V5, bilateral Optic radiation, and cerebellum (R V, L VI).

Left Occipital Temporal, Common: visual cortex (L BA17, BA18, R V4); Unique to Syntax Level: none; and Unique to Word Level: Broca's (R BA44), visual cortex (R BA 17, BA 18, L V4), bilateral premotor cortex (BA6), left corticospinal tract, R & L optic radiation, cerebellum (R & L V, L VI, vermis VI, and R V1).

Left Supramarginal, Common: somatosensory cortex (L BA1, BA2, R BA3b), secondary somatosensory cortex (L OP1, OP4), superior parietal lobule, (R & L 5M), R & L cingulum, premotor (R & L BA6); Unique to Syntax Level: superior parietal lobule (L 5ci), primary motor (L BA4a), primary somatosensory (L BA1) and Unique to Word Level: inferior parietal lobule (R PF, R & L PFM, L PFop, pFoM), superior parietal lobule (R & L 7a, L 7p), visual cortex (R & L BA17, BA18, RV5), L corticospinal tract, insula, L corticospinal tract, insula (R & L Id, Ig2), R & L cerebellum V.

Left Inferior Frontal, Common: Broca's (R & L BA44, BA45); Unique to Syntax Level: R inferior occipital-frontal fascicle; and Unique to Word Level: inferior parietal lobule (L Pga), primary auditory cortex (R TE1.0 and R & L TE1.2), parietal operculum (R OP1, OP2, L OP4), callosal body, L cingulate.

### Word versus syntax levels with affixed words

Left Precuneus, Common: inferior parietal lobule (L PF, R PFM, R & L Pga, PGp), superior parietal lobule (R 5Ci, R & L 5M, 7a, 7M, L 7p), visual cortex (R & L BA17 and BA18), callosal body, R & L cingulum, L optic radiation, R cerebellum V; Unique to Syntax Level: superior parietal lobule (R 7p), primary auditory (R TE1.2), primary motor (L BA4a), primary somatosensory (R BA1, R BA3b), secondary somatosensory; and Unique to Word Level: inferior parietal lobule (R & L Pga, R OP2, and L BA3b.

Left Occipital Temporal, Common: superior parietal lobule (R 7p), visual cortex (R BA17, L V4, V5), premotor (L BA6); Unique to Syntax Level: visual (L BA17, R V5, R & L BA18, V3V), L optic radiation, cerebellum (R & L V, VI, vermis VI, R Crus I); and Unique to Word Level: none.

Left Supramarginal, Common: inferior parietal lobule (R & L PF, L PFt), primary somatosensory (R & L BA1, BA3b, L BA2), secondary somatosensory (R & L OP1, OP4, R OP2, OP3), superior parietal lobule (R & L 5ci, 5M, 7a,7M, L 7p), R & L cingulum, visual cortex (R BA17, R & L BA8); Unique to the Syntax Level: inferior parietal lobule (R PF, L PFM, Pga, PGp), primary motor (R BA4a), anterior intraparietal sulcus (L hIP2), Broca's (L BA 44), primary auditory (R & L TE1.2); and Unique to the Word Level: superior parietal lobule (R 7p); and from the inferior frontal to R auditory cortex (TE1.2), somatosensory (R BA3b), secondary somatosensory (R OP2, OP3), premotor cortex (L BA6), R inferior occipital frontal fasciculate.

Left Inferior Frontal, Common: Broca's (R & L BA44, BA45); Unique to Syntax Level: primary auditory (L TE1.2); and Unique to the Word Level: R auditory cortex (TE1.2), somatosensory (R BA3b), secondary somatosensory (R OP2, OP3), premotor cortex (L BA6), R inferior occipital frontal fasciculate.

## Common connectivity across syntax and text levels and unique to these levels

Left Precuneus, Common: parietal lobule (R Pga, PGp), primary motor (L BA4a), superior parietal lobule (R & L 5Ci, 5M, 7a, 7M, L 7p), visual cortex (R & L BA17 and BA18) premotor (R BA6), callosal body, R & L cingulum; Unique to Text Level: superior parietal lobule (R 7p), visual cortex (L V3V, L V4, R & L V5), premotor (L BA6), Broca's (R & L BA 44 and BA45), inferior parietal (L PFM, Pga, PGp), R & L OptiC radiation, R & L cerebellum V; and Unique to Syntax Level: bilateral 5Ci in superior parietal lobule.

Left Occipital Temporal, Common: visual cortex (L BA17, BA18); Unique to Text Level: visual cortex (R BA17, BA18, R & L V5, R V4), inferior parietal lobule (L PGp), superior parietal lobule (L 7a, 7M, R & L 7p), premotor (R BA6); and Unique to Syntax Level: none.

Left Supramarginal, Common: inferior parietal lobule (L PF, PFt), primary motor (L BA4a), primary somatosensory (R & L BA1, L BA2, R BA3b), secondary somatosensory (L OP1 OP4), superior parietal lobule (L 5Ci, R & L 5M), R & L cingulum; Unique to Text--Level: primary somatosensory (L BA3a, BA3b), secondary somatosensory (R OP1, R & L OP4), superior parietal lobulue (R 5Ci, L 7a, R & L 7p), visual cortex (R & L BA17, BA18), anterior intraparietal sulcus (L hIP2 and hIP3), Broca's (L BA44, BA45), inferior parietal lobule (L PFcM, PFM, PFop, R PFt), Primary auditory (R TE1.0, L TE1.1 and TE1.2), L corticospinal tract, insula (L Ig2), R cerebellum V; and Unique to Syntax-Level: none.

Left Inferior Frontal, Common: Broca's (R & L BA44, BA45), R inferior occipital frontal fascicle; Unique to Text-Level: inferior parietal lobule (R PFOp R PFt L PGp), primary auditory (L TE1.2), secondary somatosensory (R & L OP1 OP4, L OP2, OP3), premotor (R & L BA6), callosal body, insula (L Ig2), R & L cingulum; and Unique to Syntax-Level: none.

Syntax (affixed foils) versus Text Levels. Left Precuneus, Common: inferior parietal lobule (L PF, L PFM, L Pga, R & L PGp), primary auditory (R TE1.2), primary motor (L BA4), primary somatosensory (R BA1, R BA3b, L OP2, L OP4, R & L OP1), secondary somatosensory (L OP2 OP4), superior parietal lobule (R & L 5Ci, 5M, 7a, 7M, 7p), visual (R & L BA17, BA18), premotor (L BA6), callosal body, R & L cingulum, L optic radiation, R cerebellum V. Unique to Text Level: Inferior parietal lobule (R Pga), premotor (R BA6), L cerebellum V, Broca's R & L (BA44 BA45); and Unique to Syntax Level: inferior parietal lobule (L PF), auditory cortex (R TE1.2), primary motor (L BA4a), primary somatosensory (R BA1, BA3b), inferior parietal lobule (R & L OP1, L OP2, OP4), superior parietal lobule (R & L C5i), visual cortex (R & L V3, L Optic radiation, cerebellum (R & L V, VI, vermis VI, R Crus I).

Left Occipital Temporal, Common: inferior parietal lobule (L PGp), superior parietal lobule (L 7p, R & L 5Ci, 5M), visual cortex (R & L BA17, BA18, V5, L V4), cerebellum (R & L V, VI, vermis, R & L VI, R Crus I); Unique to Text Level: superior parietal lobule (R7p, L 7a, 7M), premotor (R BA6); and Unique to Syntax Level: L optic radiation and cerebellum (R & L V, vermis, R &L VI, R Crus I).

Left Supramarginal, Common: anterior intraparietal sulcus (L hIP2), Broca's (L BA44), inferior parietal lobule (L PF, PM, PFt, Pga, PGp), primary auditory (R & L TE1.2), primary somatosensory (R & L BA1, BA3b, L BA2), secondary somatosensory (R & L OP1, OP4), visual cortex (R BA17, R & L BA18), primary motor (R & L BA4a), superior parietal lobule (R & L 5Ci, 5M, L 7p), premotor (R & L BA6), R & L cingulum; Unique to Text Level: anterior intraparietal sulcus (L IP3), Broca's (L BA45), inferior parietal lobule (R PFt), primary somatosensory (R BA3a), secondary somatosensory (L OP2), visual cortex (L BA17), superior parietal lobule (L 7a, R 7p), callosal body, L corticospinal, insula (L Ig2), R cerebellar V; and Unique to Syntax Level: inferior partietal lobule (L PFop) to primary auditory cortex (R TE1.0, TE1.2, L TE1.1), primary motor (R BA4a), secondary somatosensory (R OP2, OP3), superior parietal (R 7a, L 7m).

Left Inferior Frontal, Common: Broca's (R & L BA44, BA45), primary auditory (L TE1.2), secondary somatosensory(R & L OP4); Unique to Text Level: secondary somatosensory (R & L OP1, L OP2, OP3), inferior parietal lobule (R PFop, PFt, L PGp), premotor (R & L BA6), callosal body, R & L cingulum, R inferior occipito-frontal fascicle, insula (L Ig2); and Unique to Syntax Level: none.

## Levels of language on tasks related to hallmark dyslexia or OWL LD impairments

For each of two imaging tasks related to the hallmark deficits in dyslexia—sub-word grapheme correspondences and word-specific spelling, the dyslexia group generally, but not always, showed the same functional connectivity as the typical group), but sometimes did not.

Sub-word grapheme-phoneme judgment. The dyslexia group did not show connectivity from left occipital temporal with visual cortex (R V3V) but otherwise showed the same connectivity as the typical group had.

However, the dyslexia group showed considerable additional connectivity where the typical group did not from:

Left precuneus with Broca's area (R & L BA44, R BA45), hippocampus (L subiculum), inferior parietal lobule (R & L PF, PFM, R PFt), primary auditory cortex (R & L TE1.0, R TE1.1), primary motor cortex (R & L BA4a), secondary somatosensory (R OP1, OP4), superior parietal lobule (L 5I, R 7p), visual cortex (R & L V3V, V4, V5), premotor (R & L BA6), L acoustic radiation, fornix, R inferior occipital frontal fascicle, R & L optic radiation, insula (R & L Id1), and cerebellum (R & L I-IV, Vermis VI, R & L Crus I);

Left occipital temporal with anterior intra parietal sulcus (L hIP1, R & L hIP3), amygdala (L laterobasal group, R & L superficial group), Broca's area (L BA44 and R & L BA45), hippocampus (L cornu ammonis, entorhinal cortex, and subiculum), inferior parietal lobule (L PF, R & L PFM, R & L Pga, PGp), primary auditory cortex (R & L TE1.0), primary motor cortex (L BA4a), somatosensory cortex (L BA1, R & L BA2), secondary somatosensory cortex (R & L OP1, R OP4), superior parietal lobule (L 5I, R & L 5M, 7a, 7M, R 7p), visual cortex (R V4), premotor cortex (R & L BA6), L acoustic radiation, callosal body, R & L cingulum, R & L corticospinal tract, fornix, R & L inferior occipito-frontal fascicle, R & L optic radiation, R & L uncinate fascicle, cerebellum (R & L I-IV, L VI, R & L Crus I); left supramarginal gyrus with intraparietal sulcus (R & L hIP1, hIP2, hIP3), amygdala (R centromedial group, L superficial group), Broca's area (R & L BA44, BA 45), inferior parietal lobule (R & L PFcM, PFM, PFop, R PFt, R & L Pga), primary auditory cortex (R & L TE1.0,TE1.1, TE1.2), primary motor cortex (R BA4a), primary somatosensory cortex (R BA2, R & L BA3a, R BA3b), secondary somatosensory cortex (R & L OP2, OP3, OP4), superior parietal lobule (L 5I, R & L 7a, 7M, L 7PC, R & L 7p), visual cortex (R BA18, V4, K V5), premotor cortex (L BA6), R acoustic radiation, callosal body, R & L cingulum, R & L corticospinal tract, fornix, R & L inferior occipito frontal fascicle, R & L optic radiation, R & L uncinate fascicle, insula (R & L Id1), and cerebellum (R & L V, VI, R Crus I); and left inferior frontal with amygdala (R superficial group), Broca's area (R BA44), inferior parietal lobule (R & L PF, L PFcM, R & L PFM, Pga, PGp), primary auditory cortex (R & L TE1.0, TE1.1, TE1.2), primary motor cortex (L BA4a), primary somatosensory cortex (L BA2, BA3a, R & L BA3b), secondary somatosensory cortex (R & L OP1, OP2, OP3, OP4), superior parietal lobule (L 7a, 7p), visual cortex (R BA17, R & L BA18, L V3V, R & L V5l), premotor cortex (R & L BA6), R & L acoustic radiation, callosal body, R & L cingulum, R & L corticospinal tract, fornix, R & L inferior occipito frontal fascicle, R & L optic radiation, R uncinate fascicle, insula (L Id1, Ig1, R & L Ig2), and cerebellum (R & L V, VI, Vermis, R & L VI, R Crus I).

Lexical judgment for homonyms which are and homonym foils which are not real words. Likewise, occasionally the dyslexic group did not show connectivity where the typical group had from left supramarginal with inferior parietal lobule (R PFM) and left inferior frontal with visual cortex (R & L BA17), but otherwise showed the same connectivity as the typical group. However, again the dyslexia group showed considerable additional functional connectivity where the typical group had not from:

Left precuneus with anterior intraparietal sulcus (R & L hIP1, hIP3), amygdala (R centromedial group, laterobasal group, superficial group), Broca's area (R & L BA44, R BA45), hippocampus (R dentate gyrus), inferior parietal lobule (R & L PF, R PFcM, R & L PFM, PFt), primary auditory cortex (R & L BA4a), primary somatosensory cortex (L BA1,R BA3b), secondary somatosensory cortex (R & L OP1, OP4) superior parietal lobule (L 5Ci, R & L 5I, R 7p), visual cortex (R & L V3V, V4), premotor cortex (R & L BA6), R & L acoustic radiation, R & L corticospinal tract, fornix, R & L inferior occipito-frontal fascicle, mammillary body, R uncinate fasciculus, insula (R & L Id1, Ig2), and cerebellum (R & L I-IV, L V, Vermis VI, R VI, and R & L Crus I);

Left occipital temporal with anterior intra-parietal sulcus (R & L hIP1, L hIP2, R & L hIP3), amygdala (R & L superficial group), Broca's area (L BA44, R & L BA45), hippocampus (R & L subiculum), inferior parietal lobule (R & L PF, PFM, L PFop, R & L PFt, Pga), primary auditory cortex (R TE1.0, R & L TE1.1), primary motor area (R & L BA4a), primary somatosensory cortex (L BA1, L BA2, R BA3b), secondary somatosensory cortex (L OP1, OP4), superior parietal lobule (R & L 5 Ci, 5I, 5M, L 7a, R & L 7M), bilateral acoustic radiation, callosal body, R & L cingulum, R corticospinal tract, fornix, R & L inferior occipito-frontal fascicle, R & L uncinate fascicle, insula (L Id1, Ig2), and cerebellum (L I-IV, R & L Crus I).

Left supramarginal gyrus seed with anterior intra-parietal sulcus (R & L hIP1, R hIP2, R & L hIP3), amygdala (R & L centromedial group, R laterobasal group, superficial group), Broca's area (R BA45), hippocampus (L subiculum), inferior parietal lobule (RFop, PFt, R & L Pga, PGp), primary auditory cortex (L TE1.0, TE1.1), primary motor cortex (R & L BA4a, BA4p), primary somatosensory cortex (R BA1, BA2, R & L BA3a, R BA3b), secondary somatosensory cortex (R OP3), superior parietal lobule (R & L 5Ci, 5I, 7M, 7PC, R 7p), visual cortex (R & L V3V, V4, L V5), R & L acoustic radiation, callosal body, R corticospinal tract, fornix, R & L inferior occipital frontal fascicle, L medial geniculate body, R & L optic radiation, L superior longitudinal fascicle, R & L uncinate fascicle, insula (L Ig1), and cerebellum (R & L VI, Vermis VI, R & L Crus I); and

Left inferior frontal seed with amgydala (R centromedial group and superficial group), inferior parietal lobule (R & L PF, PFcM, PFM, L PFop, PFt, R Pga, R & L PGp), primary auditory cortex (R & L TE1.1), primary motor cortex (L BA4a, R BA4p), primary somatosensory cortex (L BA1, R & L BA2, BA3b), secondary somatosensory cortex (L OP1, OP2, R & L OP3, R OP4), superior parietal lobule (R & L 5M, 7a, L 7p), visual cortex (R V5), premotor cortex (R & L BA6), bilateral acoustic radiation, R cingulum, R & L corticospinal tract, fornix, R & L inferior occipital frontal fascicle, R & L optical radiation, R & L uncinate fascicle, insula (R & L Id1, L Ig1, R & L Ig2), and cerebellum (R VI, Crus I).

## Summary

What was not predicted and startling was the large amount of over-connectivity in that the dyslexia group showed functional connectivity with so many different brain regions where the typical group did not on both the sub-word grapheme-phoneme correspondence task and the word-specific spelling task. This pattern of results suggests that dyslexia is a disability characterized not only by impaired sub-word orthographic-phonological mapping and word reading and spelling but also by a dense personal cloud of over-connectivity that interferes with the efficiency of their written language learning. See [42] for application of the dense personal cloud to personalize medicine for purposes of prevention and treatment.

For each of two imaging tasks related to the hallmark deficits in OWL LD—reading words with true morphemes versus affix foils and sentence reading comprehension for syntax with and without affix foils—the OWL LD group showed a different pattern of results than the typical group or the dyslexia group. More often than was the case for the dyslexia group, the OWL LD group did not show connectivity where the typical group had. Sometimes, but not nearly as often as was the case for the dyslexia group, the OWL LD group showed connectivity where the typical group had not.

### Lexical judgments about words with true fixes

The OWL LD group did not show functional connectivity where the typical group had from left precuneus with inferior parietal lobule (L PF, R PFM), secondary somatosensory cortex (R OP1, OP4), L optic radiation, and cerebellum (R V); from left occipital temporal with premotor cortex (L BA6); from left supramarginal with primary somatosensory cortex (R BA1, L BA2), secondary somatosensory cortex (L OP1, R OP2, R & L OP4), superior parietal lobule (R 7a, R & L 7p), premotor (R & L BA6), and insula (R Ig2); and from left inferior frontal with Broca's area (R BA45), somatosensory cortex (R OP4), and premotor cortex (L BA6).

The OWL LD group showed the following connectivity where the typical group did not: from the left precuneus with secondary somatosensory cortex (R OP2) and insula (R & L Ig2); from the left occipital temporal with inferior parietal lobule (L PF), primary auditory cortex (R & L TE1.1), superior parietal lobule (L 7a), visual cortex (R & L BA18, L V5), callosal body, and cerebellum (R V, L VI); from the left supramarginal gyrus seed with inferior parietal lobule (R & L PFcM, L PFop); primary auditory cortex (R TE1.0, R & L TE1.1), and callosal body; and from the left inferior frontal seed, with hippocampus (L subiculum), primary auditory cortex (R & L TE1.0, TE1.1, L TE1.2), secondary somatosensory cortex (R & L OP1, L OP3), visual cortex (L BA18), R & L radiation, R uncinate fascicle, insula (R & L Id1, R Ig2), and cerebellum (R VI).

Syntactic meaning judgment of sentences with and without lexical affix foils that do or do not fit sentence context. The OWL LD group did not show connectivity where the typical group (Table 2) did from the left precuneus seed with inferior parietal lobule (L PFM), primary auditory cortex (R TE1.2), primary motor area (L BA4a, R BA1), secondary somatosensory cortex (R OP1, L OP4), superior parietal lobule (L 5Ci), and cerebellum (R V); from left occipital temporal with superior parietal lobule (L 7p) and visual cortex (R V3V and R V5); from left supramarginal with Broca's area (L BA44), primary auditory cortex (R TE1.2), primary motor area (R & L BA4a), primary somatosensory area (R & L BA1), secondary somatosensory cortex (R OP3, R & L OP4), visual cortex (R & L BA17, BA18), premotor cortex (R & L BA6), and callosal body; and from left inferior frontal with Broca's area (R BA44, BA45), primary auditory cortex (L TE1.2), and secondary somatosensory cortex (R & L OP4).

The OWL LD group showed functional connectivity where the typical group had not from left precuneus with inferior parietal lobule (R Pga), primary auditory cortex (L TE1.1), L corticospinal tract, and insula (L Ig1, Ig2); from left occipital temporal seed with inferior parietal lobule (L PFM), primary auditory cortex (R TE1.2), primary motor area (L BA4a, R BA1), secondary somatosensory cortex (R OP1, L OP4), superior parietal lobule (L 5Ci), and cerebellum (R V); left supramarginal gyrus with inferior parietal lobule (R PF), superior parietal lobule (L 5I, R 7p), and R & L corticospinal tract; and left inferior frontal seed, with visual cortex (L BA18), L cingulum, and L corticospinal tract.

## Summary

In general, OWL LD group did not always show connectivity where the typical group did whether the tasks required judgements about affixed words or syntax with affix foils, showing lower likelihood of engaging in morphological and syntactic processing, their hallmark impairments. At the same they sometimes showed more connectivity than did the typical group on reading tasks but not to the same degree as was the case for the dyslexia group. Thus, the OWL LD group showed signs of both a less and a more dense personal cloud supporting their written language learning. Both under and over connectivity at their hallmark levels of language impairment may be relevant to diagnosis and treatment of OWL LD.

## Unique connectivity across adjacent levels of language for dyslexia and OWL LD

### Dyslexia group

Comparison of unique functional connectivity at the lexical level (word-specific spellings) compared to the sublexical level (subword grapheme-phoneme correspondences) for the dyslexia group revealed a pattern not identical to that for the typical group. Connectivity from each of the four seeds that was unique to lexical (word-specific spelling—Set I) compared to sublexical (grapheme-phoneme correspondence) levels of language for the dyslexia group was as follows from left precuneus seed with L BA45, R & L optic radiation; from left occipital temporal seed with R 7a, R7p, R & L optic radiation; from left supramarginal seed with anterior intra-parietal sulcus (L hIP2), R & L BA 44, L BA45, L PFcM, L PFM, L PFop, L TE1.2, L OP2, R & L OP4, R & L BA18, L cingulum; and from left inferior frontal seed with R BA44, L Pga, R TE1.0, R & L TE1.2, R OP1, R OP2, L OP4, R & L BA18, L V5, callosal body, L cingulum.

### OWL LD group

Likewise, the connectivity across levels of adjacent language related to the hallmark deficits for OWL LD and the typical group or the dyslexia group are not identical with what was unique for the typical group or the dyslexia group. Connectivity from each of the four seeds that was unique to the lexical (affixed words—Set II) compared to syntax (with and without affix foils) levels of language was as follows from left precuneus seed with L OP1, L OP2, R 7p; left occipital temporal seed with L optic radiation, R & L cerebellum V and VI, Vermis VI, right Crus I; left supramarginal seed with none; and left inferior frontal seed with none.

## Discussion

### Evolving imaging paradigms

Current imaging methods support more fine grained detection of fMRI connectivity in the classic Broadmann areas than in the past. Quantitative results for the significant connections of greatest magnitude, after correction for multiple comparisons, can now be supplemented with profile analyses of patterns of connectivity. Both these approaches to data analyses yield insight into the nature of two common but contrasting reading disabilities—dyslexia and OWL LD. The patterns approach (results reported in text) shows that not only are specific levels of language impaired but also a personal cloud [42] of connectivity that is too dense or insufficiently connected may be contributing to persisting learning disabilities; although effective treatment has often been evaluated on the basis of normalizing BOLD activation in specific regions of interest, it may be that normalizing the density of the personal cloud^1^ supporting the complex, multi-leveled reading brain is also relevant. The more traditional reductionist approach (see Table 2) that seeks the one network of connectivity of greatest magnitude is also instructive in comparing brain connectivity across levels of language in controls and those with dyslexia or OWL LD.

### Analyses of patterns within and across levels of language in typical group

Based on descriptive patterns of connectivity within each of the four levels of language and across adjacent levels of language in Sets I and II, the first tested hypothesis was confirmed. There is a brain basis for the distinct, cascading adjacent levels of language of increasing or decreasing size, each of which draw on common as well as unique connectivity compared to levels above it or below it in both those who are typical language learners and those who have dyslexia or OWL LD. However, these patterns of common and unique connectivity are not the same in typical language learners and those with dyslexia or OWL LD. Thus, language should not be conceptualized as a homogeneous construct. Not only does language teams with different sensory input and motor output systems to create four functional language systems— language by eye, language by ear, language by mouth, and language by hand—each of these language systems has multiple levels of language. In the current study, not only levels of language—subword, word, syntax, and text—but also linguistic features in words (orthography, phonology, semantics, and or morphology) at the subword, word, and syntax levels, which were taken into account in designing tasks and drawing conclusions about levels of language, also matter.

### Analyses of patterns within and across levels of language in dyslexia and OWL LD

The second hypothesis was confirmed. Not all reading disabilities are the same in terms of the brain connectivity associated with the impaired levels of language used to diagnose them. Prior research has shown that levels of language are relevant not only to diagnosing specific learning disabilities but also to teaching children effectively to overcome their reading disabilities: Teaching to all levels of language close in time is an effective way to create a functional, multi-leveled reading system [27].

## Limitations, conclusions, and future directions

Despite the relatively small sample size, the diagnostic groups were carefully identified using evidence-based criteria and the results replicated prior research documenting BOLD activation in precuneus, occipital-temporal, parietal, and frontal regions in the reading and spelling of children with and without dyslexia [27] and connectivity from these results [28]. Recent research [7,43,44] and work in progress may shed light on how DTI white matter integrity and related fMRI connectivity may account for the anomalies in mental self-government of the complex, multi-leveled reading system observed in the current study: over-connectivity of the dyslexia group where the typical OWL group did not show connectivity; and under-connectivity of the OWL LD group where the typical OWL group showed connectivity.

## Figures and Tables

**Table 1:** Scores on Behavioral Normed Measures of Levels of Language (*z*=z score with *M*=0, *SD*=1; *DASH*, *TOC*, *CELF4* scaled scores *M*=10, *SD*=3; *TSWRF, WJ3*, *RAS*, *WISC4 M*=100, *SD*=15; see text for description).

Group	Typical (n=9)		Dyslexia (n=16)		OWL LD (n=5)	
Measure	M	SD	M	SD	M	SD
Alphabet 15z	-0.64	1.23	-1.49	0.7	-1.07 0.49	
DASH Copy Best	11.44	3.25	8.19	2.97	9.6	5.5
DASH Copy Fast	10.33	2.29	7.31	3.07	7	5.05
TSWRF	109.11	10.95	91.19	8.87	83.8	11.71
TOC Word Choice	12.26	3.59	9.13	2.73	7	1.73
Comes From z	0.3	0.29	-0.17	0.77	-0.74	0.78
TOC Letter Choice	12.67	3.04	7.25	2.08	7.4	3.05
WJ III Oral Comp	112	6	113	10.68	95.8	8.11
CELF IV Form Sent	12.78	1.92	12	2.56	6.2	1.3
WJ III Pass Comp	108.33	10.22	98.38	11.18	78	6.98
WJ III Writing Flu	107.67	7.84	92.48	10.75	83.5	13.92
WISC IV VCI	113.37	11.43	112.31	13.24	91.2	11.54

**Table 2:** Connectivity of Greatest Magnitude for Each Level of Language Task in Set I and Set II for Typical Control Group and Levels of Language Impairment in Dyslexia and OWL LD Groups.

Task	Connectivity of Greatest Magnitude	Volume (pixels)	X	Y	Z	Seed
**Grapheme-Phoneme Control**	Inferior_parietal_lobule_PGp_R	203	54	-58	24	1
	Visual_cortex_V2_BA18_R	84	4	-58	0	2
	Superior_parietal_lobule_5Ci_L	11	-12	-24	40	3
	Visual_cortex_V1_BA17_L	14	-6	-76	14	4
Dyslexia	Inferior_parietal_lobule_PGp_L	792	-44	-76	36	1
	Visual_cortex_V2_BA18_L	575	-12	-86	-2	2
	Superior_parietal_lobule_5Ci_R	228	10	-30	42	3
	Inferior_parietal_lobule_PFM_L	243	-62	-50	16	4
**Word-Specific Spelling Control**	Inferior_parietal_lobule_PGp_L	516	-46	-72	36	1
	Visual_cortex_V5_R	181	54	-62	-10	2
	Primary_auditory_cortex_TE1.2_L	16	-54	2	0	3
	Primary_auditory_cortex_TE1.2_R	48	56	-2	2	4
Dyslexia	Inferior_parietal_lobule_PGp_R	1139	44	-72	32	1
	Anterior_intra-parietal_sulcus_hIP3_R	130	32	-58	48	2
	Superior_parietal_lobule_5Ci_R	376	12	-26	42	3
	Broca's_area_BA45_L	407	-56	32	-4	4
**Syntax with/without Homonym Foil Control**	CinguluM_L	26	-4	-16	38	1
	Visual_cortex_V1_BA17_R	103	6	-84	-2	2
	Superior_parietal_lobule_5Ci_R	91	2	-28	44	3
	Broca's_area_BA44_R	62	60	12	16	4
**True/Fake Affixed Word Controls**	Inferior_parietal_lobule_PGp_R	17	52	-58	24	1
	Visual_cortex_V4_R	16	46	-72	-10	2
	Primary_motor_cortex_BA4a_L	23	-2	-22	44	3
	Broca's_area_BA44 R	68	60	12	18	4
OWL LD	none for seed 1					1
	Visual_cortex_V4_R	22	46	-66	-14	2
	CinguluM_R	41	0	14	30	3
	Broca's_area_BA45_R	26	44	44	16	4
**Syntax with/without Affixed Words Control**	Inferior_parietal_lobule_PGp_L	93	-58	-62	30	1
	Superior_parietal_lobule_7p_R	11	22	-76	56	2
	CinguluM_L	83	-6	18	34	3
	Broca's_area_BA45_R	355	54	34	20	4
OWL LD	Insula_Ig2_R	11	36	-14	14	1
	Superior_parietal_lobule_7p_R	14	10	-74	46	2
	Primary_auditory_cortex_TE1.1_R	38	40	-26	14	3
	Secondary_somatosens_Pariet_oper	37	36	-4	4	4
**Multi-Sentence**						
Control	Broca's_area_BA44_R	67	52	16	40	1
	Premotor_cortex_BA6_R	21	54	12	40	2
	Broca's_area_BA45_ L	87	-46	32	34	3
	Broca's_area_BA44_R	303	60	12	16	4

**Notes:** Connectivity seed 1 = precuneus Connectivity seed 2 = occipital-temporal Connectivity seed 3 = supramarginal Connectivity seed 4 = Broca's Area Left (inferior frontal gyrus) X Y Z are in Montreal Neurological Institute units (MNI) in mm

## Footnotes

1 Another reason for the amount of connectivity observed may be related to the astrocytes outside synaptic networks that may influence neural activity through chemical signals involving neurotransmitters and other chemical messengers [45]. That is, the human brain's connectivity may involve both wired (insulated myelinated neurons) and wireless (chemical activity outside the neurons that influence activity of the neurons).
